# Supplementary material for: Content-rich biological network constructed by mining PubMed abstracts
Source: BMC Bioinformatics. 2004 Oct 8;5:147. doi: 10.1186/1471-2105-5-147 (PMC528731; doi:10.1186/1471-2105-5-147)
Supplement: Additional File 2 — The original results of the above study (non-essential files are deleted to keep the file size under the limit set by BMC bioinformatics). [file 1471-2105-5-147-S2.bz2 › chilibotAdditionalFile2/dip30/435ID7622448E10380/html/CCL5_CCR3.html]

 


 **CCL5** and **CCR3** 
  
Found 149 abstracts in PubMed, retrieved 30.  
 

 What does Google say? 
 PDF only 
| .edu only 

---

**Interactive relationship** (e.g. stimulation, inhibition, etc)

**Stimulatory relationship**- Therefore, IL 16 activation of eosinophils is CD4 mediated to elicit the extracellular release of preformed RANTES  [ **CCL5** ]  and eotaxin, which then in an autocrine fashion act on plasma membrane  **CCR3**  receptors to stimulate both enhanced LTC 4 production and the preferential release of IL 4, but not IL 12, from within eosinophils.  Ref: 11971026 J Immunol, 2002
**Neutral relationship**- Therefore, interactions of eotaxin s, eotaxin CCL11, RANTES  [ **CCL5** ]   **CCL5** , and MCP 1 CCL2, MCP 2 CCL8, MCP 3 CCL7, MCP 4 CCL13 with  **CCR3**  are responsible for the recruitment of basophils, eosinophils and mast cells.  Ref: 12009564 Mol Immunol, 2002

**Non-interactive relationship** (e.g. studied together, co-existance, homology, etc.)

- Replacement of medium in the lower chamber the epithelial cell side with fresh medium, addition of exogenous eotaxin or RANTES  [ **CCL5** ]  in the upper chamber the gel side, or pre treatment of eosinophils with anti  **CCR3**  all inhibited transmigration.  Ref: 12047436 Clin Exp Allergy, 2002
- In two examples, we showed that chemokines influence notably the cellular immune functions, such as CD8 cytotoxicity Rantes  [ **CCL5** ]   **CCR3**  and gamma interferon production fractalkine CX3CR1.  Ref: 12134637 J Soc Biol, 2002
- The effects of IL 16 were blocked by  **CCR3**  inhibitors met RANTES  [ **CCL5** ] , anti  **CCR3**  mAb and by neutralizing anti eotaxin and anti RANTES  [ **CCL5** ]  mAbs, but not by platelet activating factor receptor antagonists CV6209, BN52021.  Ref: 11971026 J Immunol, 2002
- RESULTS Mast cells, activated eosinophils, T cells, IL 4 cells, IL 5 cells, eotaxin cells, RANTES  [ **CCL5** ]  cells and  **CCR3**  cells were all increased in the lesions of KD as compared with those in normal tissue.  Ref: 12653173 Clin Exp Allergy, 2002
- The sources of IL 4, IL 5, eotaxin and RANTES  [ **CCL5** ]  and the expression of  **CCR3**  were examined by immunostaining of serial sections with antibodies to IL 4, IL 5, eotaxin, RANTES  [ **CCL5** ]  and  **CCR3** , and antibodies to tryptase, ECP EG2 and CD3.  Ref: 12653173 Clin Exp Allergy, 2002
- We investigated tissue samples and tumor cell suspensions of patients with CD30 CTCL and CD30 CTCL for expression of the chemokine receptors  **CCR3** , CCR4, and CCR8 and the  **CCR3**  ligands eotaxin CCL11, monocyte chemoattractant protein 3 CCL7, and RANTES  [ **CCL5** ]   **CCL5** .  Ref: 12393570 Blood, 2003
- METHODS We examined the number of tryptase cells, EG2 cells, CD3 cells, IL 4 cells, IL 5 cells, eotaxin cells, RANTES  [ **CCL5** ]  cells and  **CCR3**  cells in five specimens of KD versus normal tissues by immunohistochemistry.  Ref: 12653173 Clin Exp Allergy, 2002
- Despite there being no difference in expression of CC chemokine receptor 3  [ **CCR3** ] , the priming effect of RANTES  [ **CCL5** ]  and eotaxin was significantly enhanced in eosinophils from the patients.  Ref: 12797483 Eur Respir J, 2003
- Using self organizing maps, we identified nine genes CD4,  **CCR3** , CCR5, LT beta, MIP 1 alpha, MIP 2, CD8 alpha, IP 10, and RANTES  [ **CCL5** ] , each with a unique profile of transcriptional expression, that reproduce the differentiation of states of rejection in dendrograms.  Ref: 11818457 J Leukoc Biol, 2002
- OBJECTIVE AND DESIGN The gene expression profile of  **CCR3**  ligands, eotaxin, RANTES  [ **CCL5** ] , and monocyte chemoattractant protein 3 MCP 3, was examined in normal and inflamed guinea pig lungs.  Ref: 11822789 Inflamm Res, 2001
- Matching pairs of receptors ligands were found to be coordinately expressed, including TGFbetaRII with TGFbeta3, FGFRII and cysteine rich fibroblast growth factor receptor CRF 1 with FGF7 and FGF12, CCR1,  **CCR3** , CCR5 with RANTES  [ **CCL5** ]  and calcitronin receptor like gene CALCRL with adrenomedullin.  Ref: 12584245 J Cell Sci, 2003
